# Impaired maturation of resting-state connectivity in anorexia nervosa from adolescence to adulthood: differential mechanisms of consummatory vs. anticipatory responses through a symptom provocation paradigm

**DOI:** 10.3389/fnbeh.2024.1451691

**Published:** 2024-10-24

**Authors:** Andrea Mendez-Torrijos, Mageshwar Selvakumar, Silke Kreitz, Julie Roesch, Arnd Dörfler, Georgios Paslakis, Johannes Krehbiel, Sabine Steins-Löber, Oliver Kratz, Stefanie Horndasch, Andreas Hess

**Affiliations:** ^1^Institute of Experimental and Clinical Pharmacology and Toxicology, Emil Fischer Center, University of Erlangen-Nuremberg, Erlangen, Germany; ^2^Department of Neuroradiology, University of Erlangen-Nuremberg, Erlangen, Germany; ^3^Ruhr-University Bochum, Medical Faculty, University Clinic for Psychosomatic Medicine and Psychotherapy, Luebbecke, Germany; ^4^Department of Psychosomatic Medicine and Psychotherapy, University Hospital Erlangen, Friedrich-Alexander University Erlangen-Nürnberg, Erlangen, Germany; ^5^Department of Clinical Psychology and Psychotherapy, Otto-Friedrich University of Bamberg, Bamberg, Germany; ^6^Department of Child and Adolescent Mental Health, University of Erlangen-Nuremberg, Erlangen, Germany; ^7^FAU NeW - Research Center for New Bioactive Compounds, Erlangen, Germany

**Keywords:** anorexia nervosa, food intake, brain development, fMRI, graph-theory, machine learning, snack food

## Abstract

This functional magnetic resonance imaging (fMRI) study examined resting-state (RS) connectivity in adolescent and adult patients with anorexia nervosa (AN) using symptom provocation paradigms. Differential food reward mechanisms were investigated through separate assessments of responses to food images and low-caloric/high-caloric food consumption. Thirteen young (≤ 21 years) and seventeen adult (> 21 years) patients with AN and age-matched controls underwent two stimulus-driven fMRI sessions involving RS scans before and after the presentation of food-related stimuli and food consumption. Graph theory and machine learning were used for analyzing the fMRI and clinical data. Healthy controls (HCs) showed widespread developmental changes, while young participants with AN exhibited cerebellum differences for high-calorie food. Young individuals with AN displayed increased connectivity during the consumption of potato chips compared to zucchini, with no differences in adults with AN. Multiparametric machine learning accurately distinguished young individuals with AN from healthy controls based on RS connectivity following food visual stimulation (“anticipatory”) and consumption (“consummatory”). This study highlights the differential food reward mechanisms and minimal developmental changes in RS connectivity from youth to adulthood in individuals with AN compared to healthy controls. Young individuals with AN demonstrated heightened reactivity to high-caloric foods, while adults showed decreased responsiveness, potentially due to desensitization. These findings shed light on aberrant eating behaviors in individuals with AN and contribute to our understanding of the chronicity of the disease.

## 1 Introduction

Anorexia nervosa (AN) is a severe mental disorder with one of the highest mortality rates among all psychiatric illnesses (5–6%) and a lifetime risk in women estimated to be 0.5–3% (Campbell and Peebles, 2014; Preti et al., 2009). The disorder is typically found to start in adolescents (aged 12–20 years) (Kanayama et al., 2019). It is characterized by significant weight loss accompanied by a persistent pattern of behaviors to prevent weight gain. Several neuroimaging studies have reported structural and functional brain abnormalities associated with the disorder (Friederich et al., 2013; Fuglset et al., 2016; Scharner and Stengel, 2019). Despite these findings, neural mechanisms underlying anorexia nervosa are largely unknown.

FMRI studies have used mainly stimulus-driven fMRI and symptom-provoking paradigms to examine the alterations in brain function in AN. When presented with food-related pictures, patients with AN showed increased activity in the dorsal posterior cingulate cortex, the insula, and the amygdala and reduced activity of the posterior midcingulate cortex (Gizewski et al., 2010; Horndasch et al., 2018; Joos et al., 2011), suggesting a top-down (cognitive control-related neural circuitry) dysfunction theory for AN. On the other hand, increased prefrontal activity related to food cue presentation (Hildebrandt et al., 2018) and similar findings provide evidence for the idea that cognitive-emotional top-down control affects food reward processing, possibly by overriding “bottom-up” inputs to the ventral striatum (for a review see (Steward et al., 2018)). However, the study did not focus so far on how intrinsic brain network activity might be altered due to the disorder in reaction to food cues and food consumption. During the last decade, researchers have focused their interest on spontaneous brain activity in the absence of a task or stimulus, specifically, to low-frequency fluctuations (<0.1 Hz) in the BOLD signal, known as resting-state fMRI (RS-fMRI) (Biswal et al., 1995; Lee et al., 2013). RS-fMRI searches for spontaneous fluctuations that can be correlated between brain regions that are spatially distinct (Biswal et al., 1995; Lee et al., 2013). The results are functional connectivity patterns between specific brain structures known as resting-state networks (RSNs) (Greicius et al., 2003; Lowe et al., 1998). An advantage of RS-fMRI is the ability to identify many networks simultaneously (Lee et al., 2013). Some of these functionally connected brain networks have been identified to trigger behavior (Esterman et al., 2013). Furthermore, RS-fMRI has proven to work as an early indicator of neurological and psychiatric disorders due to pathological changes in the brain (Greicius, 2008; Zhang and Raichle, 2010). Some RS-fMRI studies have demonstrated brain changes in RSNs associated with AN: In adolescent and young adult patients with AN, changes were found in the fronto-parietal network and default mode network (DMN), as well as increased functional connectivity in the anterior insula (Boehm et al., 2014). However, in another study in adults with AN, decreased connectivity was found in a visual perception network with increased co-activation in somatosensory areas (Favaro et al., 2012). Recovered patients with AN show increased coherence in the default mode network (DMN) which is thought to be involved in self-referential processing (Cowdrey et al., 2014), but in another study decreased connectivity within visual, auditory, and somatosensory RSNs (Scaife et al., 2017). These partly inconsistent results found in RS-fMRI studies may be due to the study design differences between study cohorts (Gaudio et al., 2016). First, longitudinal studies in adolescent patients before and after recovery show increased connectivity within the cortico-striatal system (CSTS) in adult and adolescent patients with a focus on the left dorsal putamen and left precuneus in adolescents (Via et al., 2021) and the left nucleus accumbens–left medial orbitofrontal cortex in adults (Uniacke et al., 2019), respectively. These alterations improved with weight restoration and symptom improvement. In another longitudinal study, widespread prefrontal, sensorimotor, parietal, temporal, precuneal, and insular reductions of resting-state connectivity were found and similarly proven to normalize over the course of recovery (Lotter et al., 2021).

Before looking further into RSN reactions toward symptom-provoking paradigms involving visual food cues and food consumption in AN, it is important to clarify how healthy responses to such paradigms work. Our early studies carried out on rats showed that specifically the mixture of 35% fat and 50% carbohydrate rather than the pure energy content in food leads to hedonic hyperphagia. This specific fat/carbohydrate ratio can be found in high-caloric snack foods such as potato chips. Furthermore, Schulte et al. (2015) assessed among 518 participants a list of 35 foods and their likelihood to be addictive. Of those, potato chips were found to be the third most addictive food. Our previous study in healthy adults showed that viewing followed by ingestion of high-calorie (potato chips) vs. low-calorie food types was able to generate different changes in RSNs. Furthermore, in healthy participants, we found that BMI positively correlated with nucleus accumbens activity when consuming potato chips, pointing toward a significance of this brain region that has been shown to be affected in AN in motivational processes related to food consumption (Mendez-Torrijos et al., 2018). Following these findings, we aimed to extend our research to clinical populations of AN. It is crucial to consider the involvement of differential food reward systems in the brain, as abnormal responses to food in AN were found to be more driven by altered motivational salience (“anticipatory”) than by explicit “consummatory” responses (Cowdrey et al., 2013). These two different mechanisms are necessary to experience reward completely and have been proven to have distinct brain connectivity patterns (Berridge, 1996; Rolls, 2012; Simon et al., 2017). On the other hand, inhibitory control networks involving mainly (pre-)frontal but also a range of other brain areas could be of interest in AN (Kang et al., 2022). Overall, a deeper understanding of the neuronal processes involved in the perception and processing of food stimuli would contribute to a better understanding of AN and, consequently, to the development of better pathogenetic models and therapy concepts. In addition, only a small number of studies include adolescents in their sample. Adolescents have a higher risk of developing an eating disorder due to a number of different environmental, social, psychological, and biological factors. Given the significance of this early onset of AN, it is crucial to include adolescents/young adults in AN studies to clarify the etiology and course of this disease.

The aim of this study was to contribute to the understanding of the underlying neuronal processes of food-related information processing in AN from adolescence into adulthood. For this purpose, patients with AN and healthy controls (HCs), each in two different age groups (young: ≤21 years and adult: >21 years), were examined in a symptom provocation experimental design including the presentation of disorder-specific stimuli to assess “anticipatory” responses and food consumption to assess “consummatory” responses. Thus, the role of motivational and affective eating processes was differentiated by RS-fMRI measurements.

**Figure 1:**
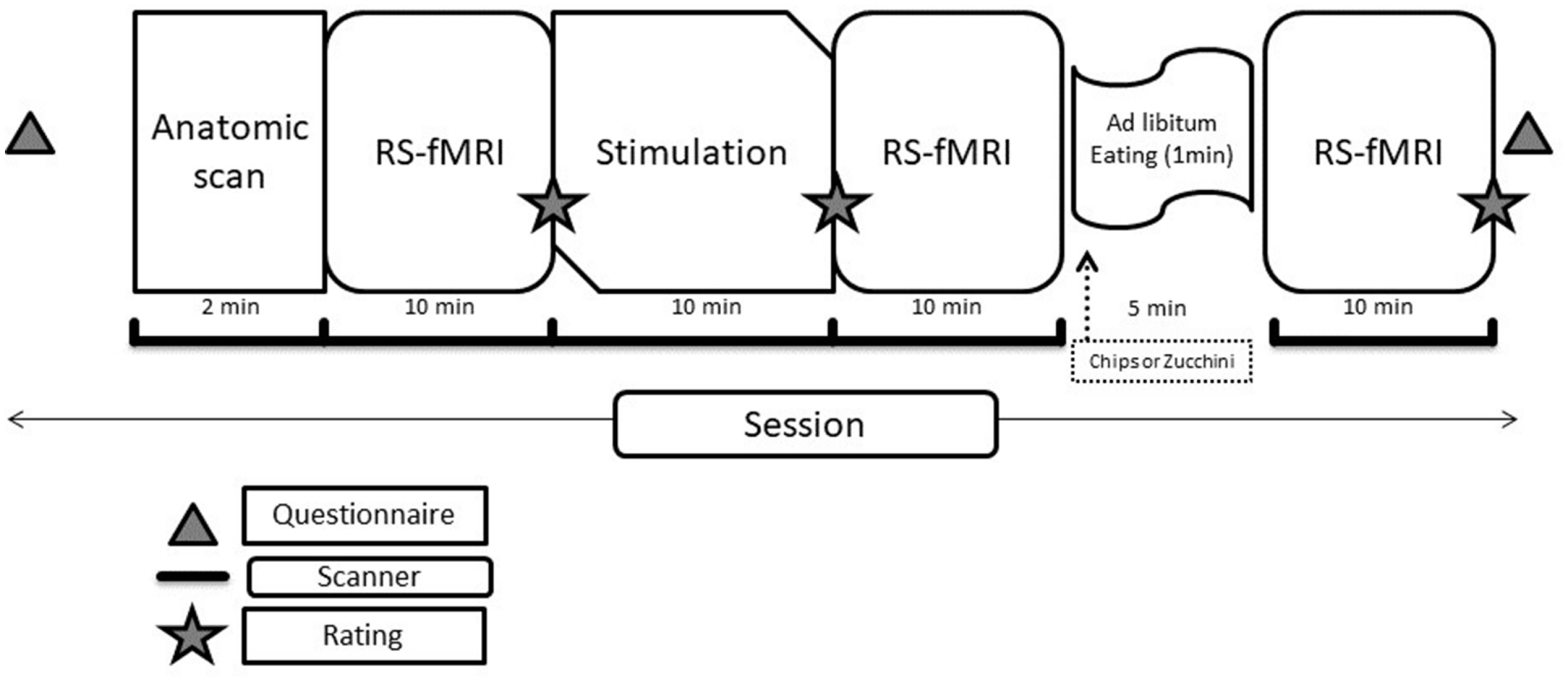
Schematic of the study protocol.

The following questions were assessed for each of the experimental stages (see Figure 1):

First, the study sought to determine whether there are RS connectivity alterations in the brains of patients with AN compared to HCs and whether developmental differences in RS connectivity exist between these groups. This was done via the first RS-fMRI scan, referred to as baseline due to the lack of previous stimulation.

Additionally, the impact of visual stimulation on RSNs was investigated to see whether it differentially affects AN vs. HC participants. This focused on the differences between the baseline and the second RS-fMRI scan.

Finally, the study also explored how food consumption influences RSNs in AN, specifically examining whether the intake of low-calorie and high-calorie foods has different effects. Furthermore, it aimed to identify which brain structures respond selectively to the consumption of high vs. low-calorie foods. This last objective focused on the third RS-fMRI scan, which followed the food consumption phase.

## 2 Methods

### 2.1 Participants

Participants were individuals with AN or HCs. Eligible patients for young and adult groups were females and between 12 and 41 years old. The participants with AN were recruited during inpatient treatment at the University Clinic Erlangen and fulfilled the diagnostic criteria of AN (ICD-10: F50.0 or F50.1), diagnosed by an experienced (child) psychiatrist or psychologist using the “Kinder-DIPS” diagnostic interview (Schneider et al., 2017) in adolescents and the Structured Diagnostic Interview for Mental Disorders (Suppiger et al., 2009) in adults. HCs were included if they had a normal BMI (18–25 kg/m^2^) and no previous history of an eating disorder. All individuals were excluded if they suffered from chronic physical diseases, especially cardiovascular and neurological diseases, had any MRI contraindications, a current pregnancy, profound developmental disorders, intellectual disabilities, or psychotic disorders. Anxiety and depressive disorders were not exclusionary, as these disorders share high comorbidity with AN. The study was open to all qualified volunteers throughout its 3-year duration. It should be noted that compliance, in particular with young patients, needing parental approval, is not very high. Participants were group-matched by age at the first MRI measurement, resulting in two groups: young: ≤21 years (developing brain) and adult: >21 years (mature brain).

Seven participants were excluded: one due to excessive head movement (> 3.5 mm which can lead to brain structure misidentification, particularly for small subcortical structures); one due to scanning corruption; and five dropouts during the measurements (three adults (all AN) and two young (1 HC, 1 AN)). All participants with AN corresponded to the restrictive type diagnosis.

The final sample consisted of 15 adult HCs, 13 adults with AN, 16 young HCs, and 17 young individuals with AN.

All participants gave written informed consent, the Ethics Committee of the University Clinic Erlangen approved the study, and all research activities were conducted in accordance with the Declaration of Helsinki (2013).

### 2.2 Magnetic resonance imaging acquisition

The MRI data were collected at a 3 T scanner (Magnetom Trio; Siemens) using a standard 12-channel, phased-array head coil: for anatomic datasets, a T1-weighted, magnetization-prepared, rapid gradient-echo (standard Siemens MPRAGE: TR = 1900 ms, TE = 2.52 ms, matrix: 256×256, FOV = 256 mm x256 mm, voxel resolution = 1 mm x 1 mm, 176 slices, and slice thickness = 1 mm). Functional RS data were acquired using a standard single-shot, echo-planar imaging (EPI) sequence of 200 volumes with each 36 slices (TR/TE = 3000/30 ms) using a 128 × 128 matrix resulting in a spatial resolution of 1.5 mm in plane, 3 mm slice thickness, and 0.75 mm gap between slices.

### 2.3 Study design

The current study is designed as a non-randomized, controlled clinical-experimental study. The study included three dates (D1 to D3), one pre-measurement and two experimental measurements, the latter separated by 1 week.

**Figure 2:**
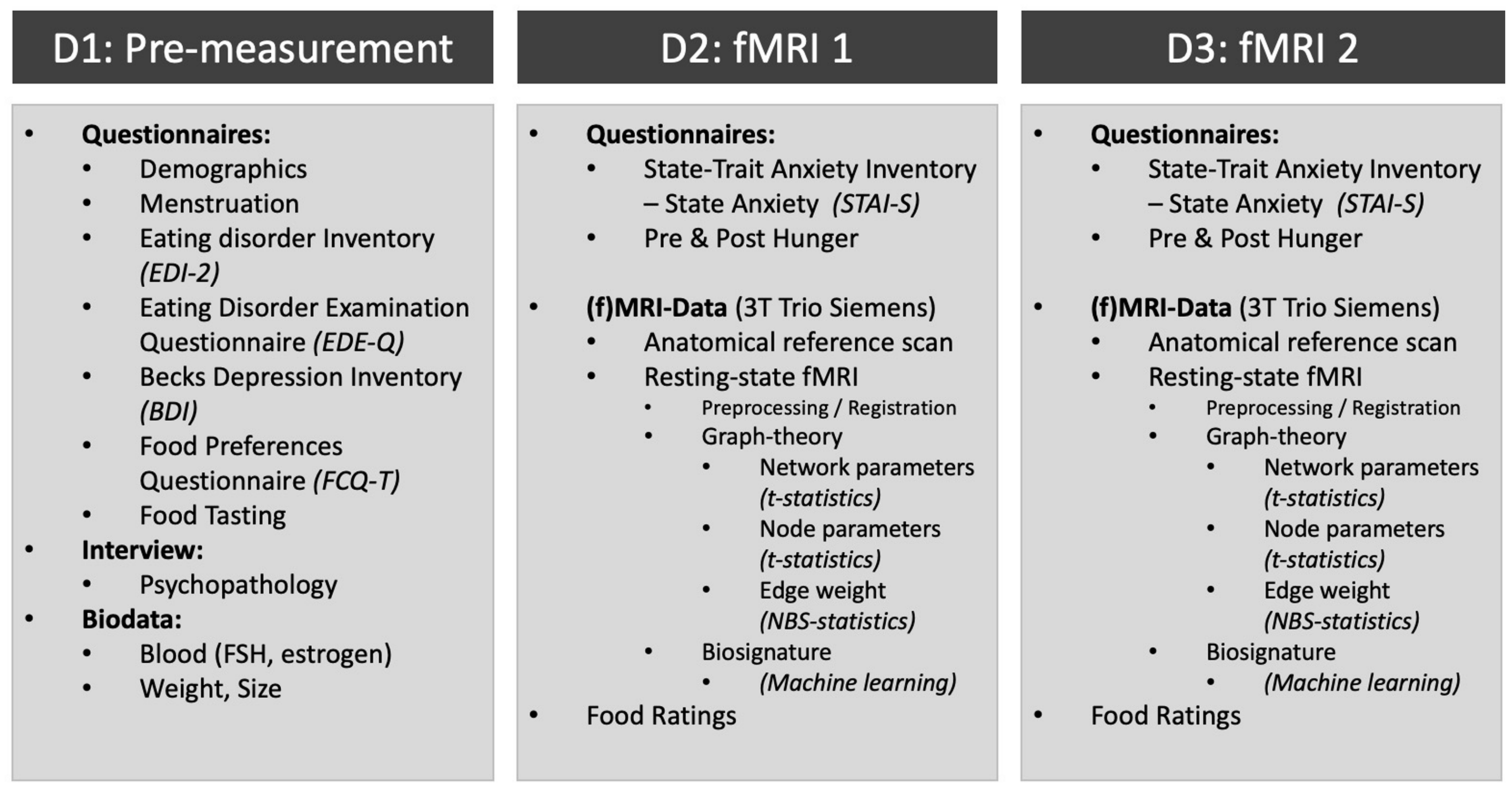
Schematic of the assessment schedule and acquired data.

During D1 (duration approximately 1 h), participants were asked to taste the test food: potato chips brand “funny-frisch® salted chips” (528 kcal/100 g, 33% fats, 49% CHO), raw zucchini slices (17 kcal/100 g, 3% fats, 3.5% CHO), and raw carrot slices (41/kcal, 2% CHO, 9.6% fat). Zucchini was chosen as the test food because it is one of the vegetables with the lowest caloric content (Tejada et al., 2020) and has a mild, neutral taste as opposed to more savory vegetables and therefore serves as a control food. The subjects were informed that they would eat two of these three foods during the measurements. Carrots were not administered, but shown for distraction purposes (participants could not foresee what food they would receive as this might have had an unwanted effect at the second scan). Then, a psychopathological interview was carried out by a researcher trained in psychodiagnostics. Participants filled in several questionnaires (see Figure 2), their height and weight were measured, and a blood sample was taken by a physician to examine endocrine values.

The MRI measurements (D2 and D3) took place at the same time (4–5 pm) each week. Participants attended half an hour before the MRI measurement and received a standardized snack consisting of 200 mL of orange juice and 70 g Brandt® rusk “Snack-Pack” to ensure that all participants had about the same state of satiation. Their current fatigue, anxiety (STAI-S), hunger sensation, and possible sleep disturbances were noted. At the end of each measurement, their current hunger and momentary anxiety were registered again.

Each fMRI session (sequence protocols in Figure 1) had a length of ∼50 min in total. The experimental software Presentation® Version 20.2 (Neurobehavioral Systems Inc., Berkeley, CA) was used for stimulus presentation and response acquisition. The images were presented through a projector, which the subjects saw through a mirror located above their heads. The subjects gave the answers to the assessments of the stimuli within the MR scanner via response grips (NordicNeuroLab AS, Bergen, Norway).

Each fMRI session started by acquiring the individual T1-anatomy. Then, the participants were instructed to keep their eyes open, and the first RS-fMRI was measured. The stimulation/priming scan was a block design with 16 blocks. Each block (18 s) was composed of six images of the same stimulus category (chips, zucchini, carrots, and colorful wooden blocks). Wooden blocks were used as a visual control condition; furthermore, all pictures of high-and low-calorie food were matched according to color, image density, and area (in percentage of the total picture) occupied by the (food) item. At the end of each block, a fixation cross was presented (18 s). Before and after the stimulation, the participants were instructed to rate on a scale from 0 to 9 their desire to eat chips, zucchini, and carrots (0 = none, 9 = very strong). The stimulation and second rating were followed by the second RS scan. There was then a pause of 5 min, during which the participants were moved out of the scanner but remained on the motor table and consumed chips or zucchini slices (the session in which each participant received one or the other was randomized) *ad libitum* for 2 min with a minimum amount of 5 g of chips and 20 g of zucchini. They were then moved back into the scanner for the third RS scan and a last rating [Note: The results of the BOLD stimulation are beyond the scope of the current publication].

### 2.4 Data processing and analysis

The RS-fMRI data were analyzed for each subject, session, and measurement. The preprocessing of functional data in BrainVoyager QX® (V. 2.8, Brain Innovation B.V. Maastricht, Netherlands) included slice scan time (sinc interpolation with TR = 3,000 ms and appropriate slice ordering) and head-motion correction (3D trilinear with sinc interpolation) in order to detect and correct small head movements by spatially aligning all volumes to the first volume through rigid body transformations (Goebel et al., 2006). Due to software limitations of BrainVoyager®, MagnAn® (BioCom GbR, Uttenreuth, Germany) was used to regress out the white matter and ventricle signals and next to perform a band-pass filter (0.009 Hz. to 0.08 Hz) followed by a 3D spatial smoothing with FWHM = 3 mm. T1 and the average RS volume (the average volume of all time points of each RS serves as an anatomical reference for the respective functional scan) were skull-stripped using the HD-BET tool (Isensee et al., 2019). Afterward, each low-resolution EPI BOLD dataset was registered using ANTS (ANTS; Penn Image Computing and Science Lab) in two steps to the MNI space (first an affine registration to the T1 anatomical image and then a symmetric diffeomorphic image registration to the MNI space for the T1 anatomical images); the registration process generates the transformation files from one space to the other, which were used in reverse to transform our in-house optimized probability atlas from the MNI space to the BOLD image subject space, and doing so segmenting the low-res bold images. For our study, we designed an optimal MNI-based probability atlas combining different existing parcellation schemes based on the Harvard Oxford Atlas (Desikan et al., 2006; Frazier et al., 2005), arousal system critical to consciousness and disorders (Edlow et al., 2012), and hypothalamic sub-parcellation (Makris et al., 2013) to complete 205 brain regions relevant for eating disorders. This atlas was used to define the different regions of interest (ROIs) necessary for the multi-seed region analysis, which preceded graph theory.

### 2.5 Functional connectivity analysis via graph theoretical method

To explore connectivity differences across brain regions we used the multi-seed region analysis approach (MSRA) (Kreitz et al., 2018), which relies on multiple seed correlation maps: the mean time course of a seed region per brain region was correlated with the time course of every voxel in the brain resulting in one correlation volume per brain region. Seed regions (six voxels) were determined automatically through the center of mass per atlas brain region. Significant correlations within that correlation volume were determined using a false discovery rate (FDR, q = 0.05). For each seed region, the significant correlation values were averaged per atlas brain region, resulting in a 200 × 200 asymmetric correlation matrix. This matrix could also be represented as network graphs consisting of vertices (or nodes) and edges. Vertices represent the structures of the brain, whereas edges between pairs of brain regions indicate their functional connectivity.

Network metrics were derived from the binarized matrices using MagnAn. Global graph metrics quantify the network’s properties of integration and segregation and, therefore, are suitable to describe the efficiency of information flow within a network. Weighted density thresholded networks per subject measurement and RS were characterized using the following graph theoretical global network measures for a density range from 2 to 20% of all possible connections: the clustering coefficient *γ* and the average path length, *λ* both normalized to 100 random networks of the same size, and the small world index *σ*, i.e., the quotient of g and l (Watts and Strogatz, 1998). In case of σ, this resulted in a hyperbolic curve (*cf.* Supplementary Figure S1). As the topology of a network graph is strongly dependent on the number of represented connections, we chose the density in the turning point of the obtained s-curve (7%) for further graph theoretical analysis and statistics between networks (*cf.* Supplementary Figure S1). At this density, the graph is highly interconnected but sparse enough to be distinguishable from a random network. Additionally, the node-specific node graph theoretical parameters strength, degree, and hub scores (Watts and Strogatz, 1998) were calculated.

Comparisons of demographic and clinical characteristics and ratings for food desire during measurements between each cohort for their corresponding age group were performed on SPSS (IBM SPSS Statistics for Macintosh, Version 25.0., Armonk, NY: IBM Corp) using the independent two-sample *t*-test, with *p* < 0.05 considered significant.

Significant connectivity differences, i.e., topology differences, between average group networks based on connectivity strength per subject were determined using network-based statistics (NBS) first introduced by Zalesky et al. (2010) (Supplementary Table S1). NBS is sensitive for detecting effects in spatially extended networks of altered connectivity, therefore accounting for mutual and dynamic interactions between brain regions. To test for the different types of hypotheses, we performed two different types of NBS, paired and homoscedastic testing.

For homoscedastic NBS the largest component of altered connections was determined using homoscedastic *t*-statistics at a significance level of *α* = 0.05 and controlled for the family-wise error (FWE) using permutation testing with 10,000 random permutations of the subject between groups.

However, when the hypotheses implied testing for paired design groups, permutations cannot be used. To overcome this limitation for some hypotheses, we implemented a modified version of the network-based statistics first described by Kreitz et al. (2018), where an additional control experiment consisting of the same patients without experimental stimulation is included. The control experiment is used to adjust alpha, to test for significant modulations specific to the experimental group. In this study, we examined the influence of food consumption between the second and third resting-state measurements (*cf.* study design, Figure 2). During the control experiment, the participant ate zucchini in between both resting-state measurements, during the stimulated experiment chips. The control experiment served to rule out the effects of interrupting the fMRI session, thereby highlighting the effect of junk food (i.e., chips) consumption.

The different contrasts and resulting statistical values are shown in Supplementary Table S1. The resulting networks were represented using Amira 5.4 (Thermo Fisher) using custom-made modules.

### 2.6 Multiparametric machine-learning analysis

Modern technologies provide high-dimensional data spaces that are hard to analyze with classical statistics (e.g., multiple comparison problems and curse of dimensionality). Datasets with small sample sizes and a few dozen input parameters were the basis for designing classical statistics. However, with high-dimensional datasets, the number of input variables increases, increasing their possible associations. As a result, these relationships are more complex, consequently leading to statistical inferences that are less precise (Bzdok et al., 2018) or combinatorial relationships might be overlooked. Therefore, we employed a dedicated machine-learning (ML)-based analysis framework to assess the resting-state data with all available global graph metrics and node-specific parameters. The framework extracts, purely data-driven, the most notable feature combinations, i.e., RS-biosignature, that indicate separations between the different experimental groups and conditions.

Our dataset comprised a total of 6,272 features, i.e., a combination of brain structure and measured value, derived from two atlases: GraphInfo with 266 parameters and NodeParams with 2,870 features, for each of the two resting states (RS2 and RS3). The ML algorithms were provided with GraphInfo parameters across a range of network densities from 0.02 to 0.2. This approach allowed a comprehensive view of network properties at different levels of brain connectivity.

Training data comprised 75%, while 25% were used as test datasets and cross-validated 100 times. For additional algorithmic cross-validation and to emphasize robustness and reliability, we used (a) two different feature selection (FS) approaches [Boruta (Kursa et al., 2010; Kursa and Rudnicki, 2010) and sparse partial least squares discriminant analysis (sPLS-DA) (Cao et al., 2011)] both using XGBoost classifier (Chen and Guestrin, 2016) which provided best results. This approach aided in acknowledging the fact that all models do not work well on all datasets and also cross-validating the results across models. The FS algorithms aided in filtering out the most relevant features from the high-dimensional dataset comprising the spectrum of network densities and network parameters.

The whole pipeline was built on R, and various packages [Boruta (Kursa et al., 2010), RandomForest, Caret, and mixOmics (Rohart et al., 2017) among others] were used for FS and classification.

## 3 Results

### 3.1 Sample characteristics

The demographic and clinical characteristics of the final sample are summarized in Table 1. There were no significant differences in age between the cohorts for each age group. The AN sample showed overall significantly lower BMI and higher BDI, FCQ, EDQ, and EDI-II scores. On the other hand, results for the STAI-S showed that the AN cohort had lower state anxiety before and after the scan than the control participants with significant differences between the groups except for the post-questionnaire between the younger groups. Furthermore, these STAI-S ratings before and after measurements were constant for each group. FSH was found to not differ significantly between the two groups. Estradiol was only analyzed for values above 5.0 (pg/mL); lower values were not registered. Nevertheless, a tendency toward lower values in AN was shown.

**Table 1:** Demographic characteristics and clinical data of the included sample.

	Adult (> 21 years)					Young (≤21 years)				
	HC (*N* = 15)		AN (*N* = 13)			HC (*N* = 16)		AN (*N* = 17)		
	*M*	(SD)	*M*	(SD)	*p*	*M*	(SD)	*M*	(SD)	*p*
Age (years)	25.2	(4.3)	25.1	(3.5)	0.89	17.3	(2.5)	16.8	(3.0)	0.57
BMI* (kg/m2)	23.5	(3.5)	17.1	(1.8)	**<0.001**					
BMI–Age percentile						56.8	(22.6)	7.4	(7.8)	**<0.001**
Education Duration (years)	11.7	(1.9)	11.1	(1.3)	0.46	8.5	(2.1)	7.9	(2.5)	0.27
FSH (IU/mL)	3.9	(2.9)	3.4	(2.8)	0.65	3.5	(2.3)	4.6	(2.5)	0.25
Estradiol (pg/mL)	63.6	(9.2)	21.8	(2.0)	0.11	68.5	(8.4)	22.0	(3.3)	0.06
BDI	4.9	(5.8)	24.2	(9.3)	**<0.001**	5.9	(5.1)	28.4	(12.1)	**<0.001**
FCQ-T	27.0	(10.6)	44.1	(15.9)	**0.002**	25.4	(10.3)	46.1	(19.7)	**0.001**
EDE-Q **	0.63	(0.35)	3.27	(1.26)	**<0.001**	0.56	(0.58)	3.89	(1.5)	**<0.001**
EDI-II	141.7	(30.5)	225.1	(30.6)	**<0.001**	131.8	(26.4)	245.9	(42.1)	**<0.001**
STAI-S pre***	43.0	(3.3)	37.9	(4.3)	**0.002**	42.8	(3.1)	38.5	(4.0)	**0.002**
STAI-S post***	43.1	(4.9)	37.86	(4.8)	**0.008**	41.2	(4.1)	39.1	(2.1)	0.18

HC, healthy control; AN, anorexia nervosa; SD, standard deviation; N, numbers; BMI, body mass index; FSH, follicle-stimulating hormone; BDI, Beck’s Depression Inventory; FCQ-T, Food Cravings Questionnaire—Trait; EDE-Q, Eating Disorder Examination—Questionnaire; EDI-II, Eating Disorder Inventory-2; STAI-G, State Trait Anxiety Inventory–State (pre = before MRI, post = after MRI). Independent *t*-test applied, Levene’s test for equality of variances, two-tailed *p* provided. *BMI for the young age group was calculated using the corresponding BMI-for-age percentile based on Kromeyer-Hauschild (Kromeyer-Hauschild et al., 2001). **Two separate versions (adult and youth) of the EDQ were applied to their corresponding age group. ***Show the average STAI-S results for the two measurements as there was no significance found across them. Bold values: significance *p* < 0.05.

**Figure 3:**
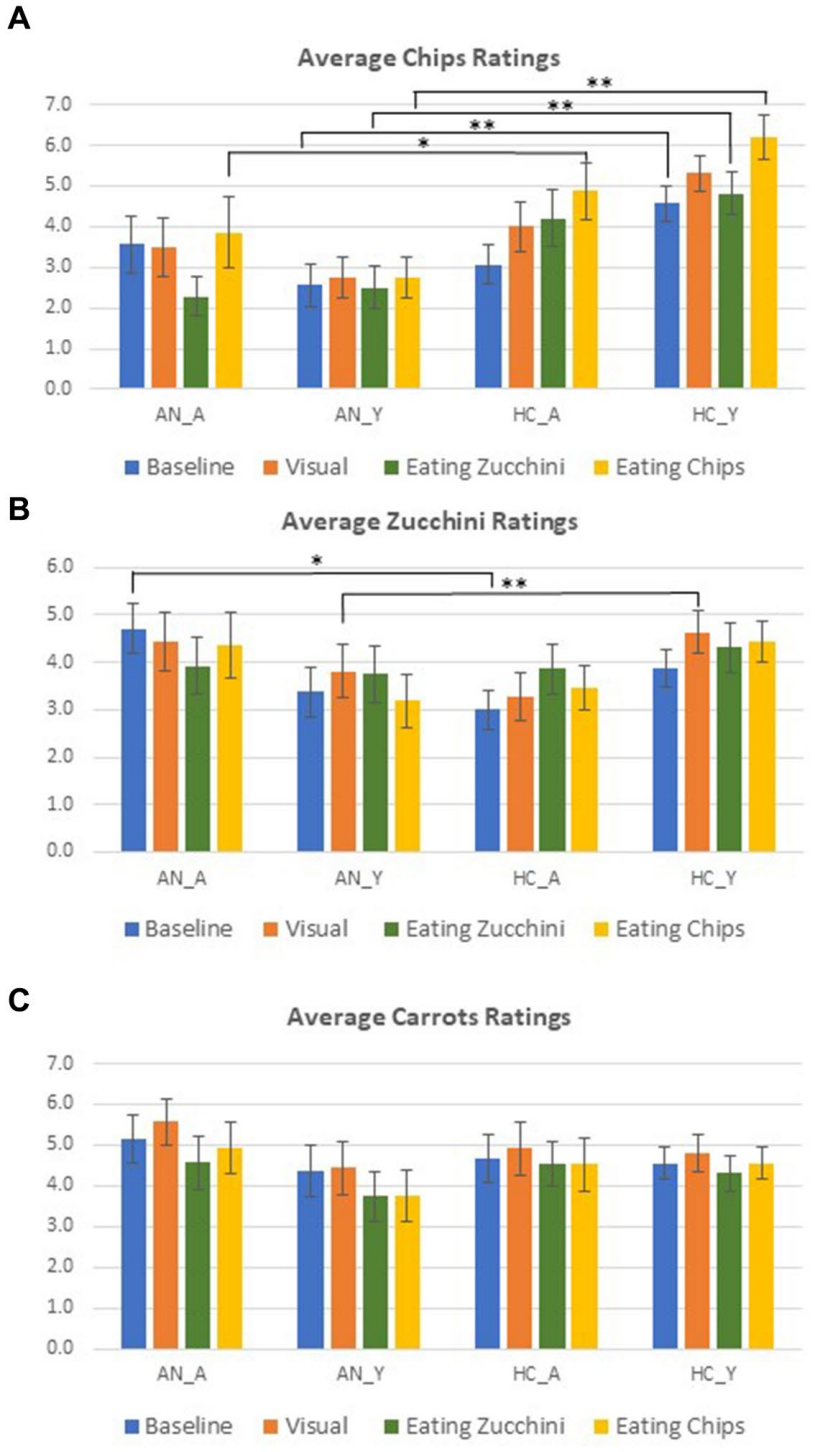
The response rate for food desire ratings during measurements: **(A)** chips; **(B)** Zucchini; **(C)** Carrots. Error bars represent standard error. The average and standard error of response rate for the different ratings that took place across the two measurements are presented. Independent *t*-test applied, Levene’s test for equality of variances, two-tailed p provided; *t*-tests were calculated between each cohort for their corresponding age group (* = *p* < 0.05, ** = *p* < 0.01).

Results from ratings (food desire) shown in Figure 3 revealed significant differences solely between AN and HCs for young participants at baseline (*p* = 0.01), after visual stimulation (*p* < 0.001), and after food consumption (for chips and zucchini *p* < 0.001), with significantly higher ratings for HCs. This effect was not found among adults with the exception of the chips ratings after consuming chips, where HCs rated once again significantly higher than AN (*p* = 0.03). Results also showed significantly higher ratings for adults with AN than HCs for baseline zucchini ratings (*p* = 0.01) and young HCs compared with AN after the visual stimulation (*p* < 0.001). Of note, the small error measures in Figure 3 indicate low individual preferences. Overall, AN responded relatively similarly in their ratings across the three RS and did not variate their responses according to the different types of stimulation and reported less explicit anticipatory responses for high-calorie food than for the two low-calorie conditions. On the other hand, HCs increased their overall ratings after stimulation when compared to the baseline (*cf.* Figure 3).

### 3.2 Connectivity analysis

Overall, the general global RSN connectivity as indicated by the normalized clustering coefficient, normalized average shortest path length, and small world index did not differ across the cohorts and age groups for the different RS (Supplementary Figure S1). The small world values ranged between 2 and 4 at high densities above 10% and were in the normal range of adult human resting-state networks. Thus, the overall efficacy of information flow within the RSN given by those measures was unaffected.

**Figure 4:**
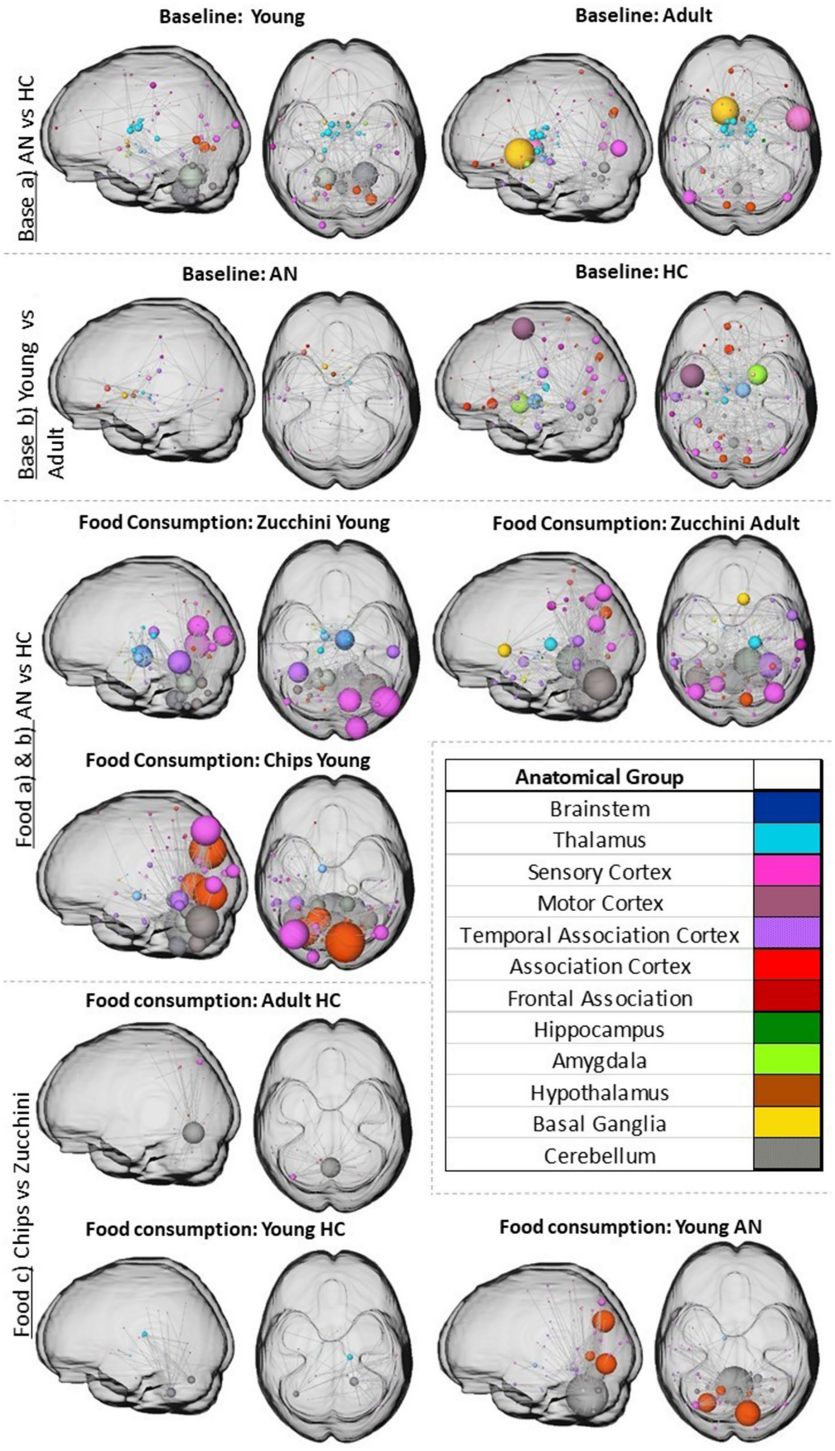
The figure shows the increased significant network differences for the different hypotheses identified by NBS (for *p* and alpha values check Supplementary Table S1). The nodes and the edges are overlaid with an MNI glass brain in two different planes (axial and sagittal). Node size represents the total degree (total amount of significant connections with the rest of the network).

The resulting values for NBS according to our hypotheses are shown in Supplementary Table S1. In consistent with our previous publication where we found stable RSNs across measurements (Mendez-Torrijos et al., 2018), we focused on measurement one for our analyses. The resulting networks are displayed in Figure 4. NBS revealed that young participants (AN vs. HCs) present larger baseline RS connectivity differences in the cerebellum and adults (AN vs. HCs) in the sensory cortex and basal ganglia (Figure 4A). In both age groups, the RS connectivity in the thalamus was also significantly increased. Developmental changes (differences between young and adult groups) result in more widespread connectivity differences between both HC age groups than in those with AN (Figure 4B). Furthermore, AN showed a high pFWE of 0.49 (Supplementary Table S1), indicating a high probability that the few developmental differences in this group are random. For HCs, the thalamus, motor cortex, and amygdala displayed significant connectivity differences between the younger and the older groups.

The NBS of the visual stimulation effects on RS networks was not carried out given that the alpha calculated using HCs as control was too low; therefore, no different connections for AN could be observed for that specific alpha. The reason for that usually is that the control group has more significant differences than the experimental group.

The resulting networks for the different food items consumed (Figure 4C) showed overall significant differences (NBS with *α* = 0.05, FWE) for all conditions in the cerebellum, sensory, and association cortex. Adult HCs vs. AN participants showed no significant differences for chips. Young participants showed more significant differences between individuals with AN and HCs in the visual cortex and even stronger in the cerebellum for the condition of the chips. Interestingly, both young groups showed differences in the substantia nigra for both food items and in the parabrachial pigmented nucleus for zucchini. The adult group showed additional thalamic, middle temporal, and parahippocampal gyrus differences between individuals with AN and HCs for the zucchini condition.

When checking which RS network brain structures selectively respond to high-vs. low-calorie food consumption (Figure 4D) results showed predominantly significantly increased connectivity after eating chips vs. zucchini for young individuals with AN in the cerebellum and association cortex, specifically in the cuneus and occipital areas. However, adults with AN had no significant differences. Young HCs showed less striking significant differences and similar patterns to adult HCs.

### 3.3 Machine learning analysis

Beyond classical parameter statistical assessments, we used ML on RS2 (“anticipatory”) and RS3 (“consummatory”) data to establish a biosignature of parameter combinations that lead to the separation of AN from HCs. We found no separation for the adult group. For the young group, AN could be separated from HCs using RS after visual stimulation (RS2) compared to RS after food consumption (RS3).

**Figure 5:**
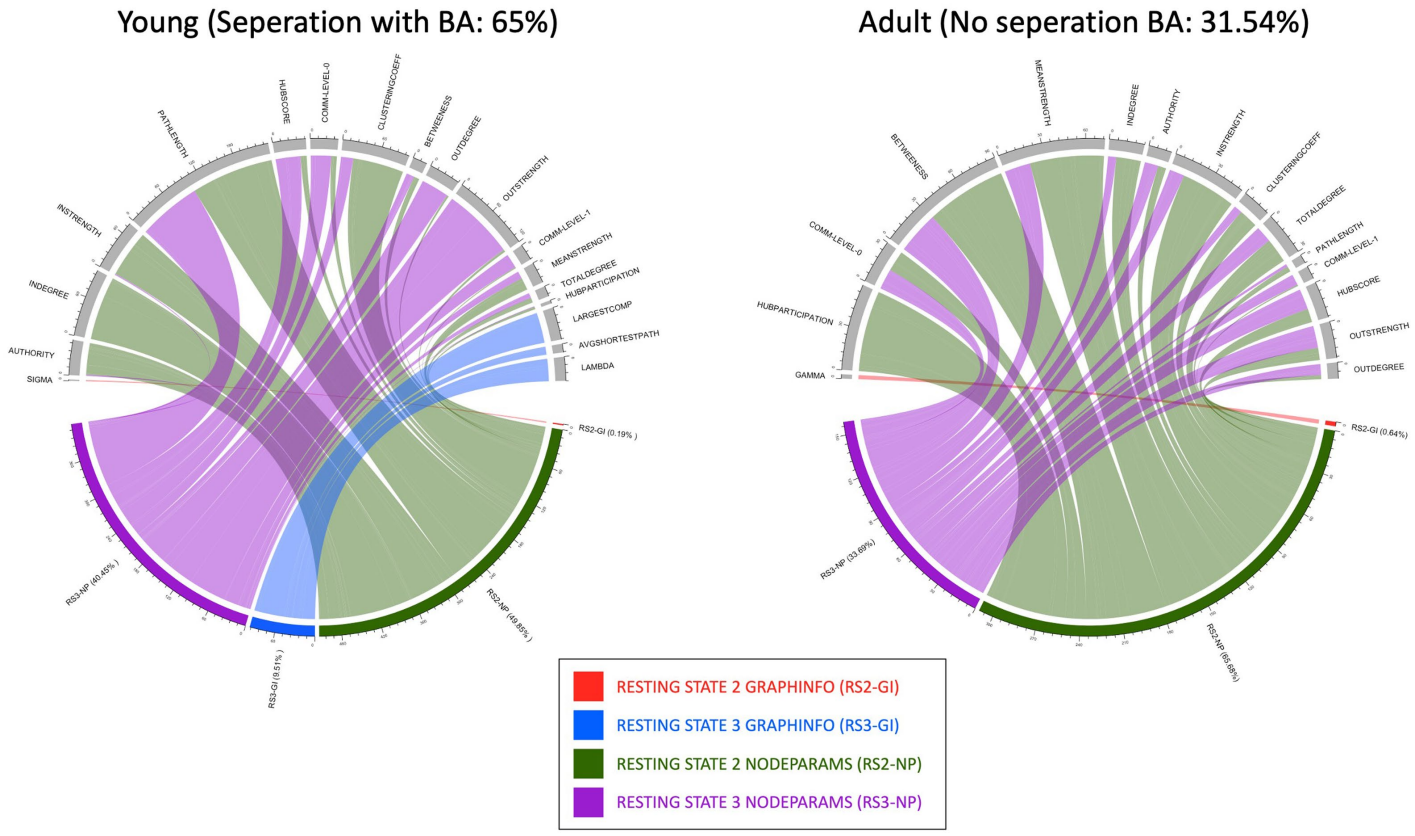
This figure shows the different chord plots containing the distribution of the features selected by the Boruta algorithm classifying anorexia to controls, with the feature counts representing the sum over 100 iterations. The plots show **(A)** the young RS data comprising of measurements 2 and 3 with a sensitivity: 72.25%; specificity: 57.75%; balance accuracy: 65% and **(B)** the adult RS data comprising of measurements 2 and 3 with a sensitivity: 33.83%; specificity: 29.25%; balanced accuracy: 31.54% meaning no separation found. In the lower semicircle, we see the atlases GraphInfo (GI) and Nodeparams (NP) with their relative percentages and distribution to the various measures on the upper semicircle. The percentages shown represent the proportion of times each feature was selected across 100 runs, with 100% indicating consistent selection in all iterations.

Both FS algorithms consistently separated AN from HCs in the young group. The number of features selected by each algorithm on different age groups is summarized in Table 2. As separation in the young group was better with the Boruta FS algorithm together with the XGBoost classifier vs. sPLS-DA (Table 2), we continued using the Boruta algorithm for further analysis. In addition to this, Boruta identified a more streamlined set of features that required less extensive hyperparameter optimization, while at the same time performing better or similar to sPLS-DA. Figure 5 shows the distributions of the selected features of Boruta algorithm (for top features, ref. Table 3) as chord plots for the young and adult datasets. The figure shows that RS2 and RS3 node parameters (NP) (shown in green and purple, respectively) dominate the separation and are relatively equal in their contribution. For both the datasets, adult and young, RS2 NP is more frequent than others (Figure 5). It is important to note that the number of features represented in these chord plots is the sum of over 100 iterations of the algorithm, providing a comprehensive view of feature importance across multiple runs. The percentages displayed represent the frequency of feature selection across 100 iterations, where 100% indicates consistent selection in all runs.

**Table 2:** Performance comparison of feature selection algorithms and classifiers on adult and young RS2 and RS3 datasets.

Data (RS2 + RS3)	FS algorithm	Features selected	Classifier	Balanced accuracy	Sensitivity	Specificity
Adult data	Boruta	5	XGBoost	31,54%	33,83%	29,25%
Adult data	sPLS-DA	20	XGBoost	42,08%	40,25%	43,92%
Young data	Boruta	10	XGBoost	65%	72,25%	57,75%
Young data	sPLS-DA	20	XGBoost	63,46%	60,25%	66,67%

The table shows balanced accuracy, sensitivity, and specificity for combinations of Boruta and sPLS-DA feature selection with the XGBoost classifier. All metrics represent means over 100 iterations, with young data models generally outperforming adult data models.

**Table 3:** Most relevant features (present in over 50% of the iterations) from the Boruta algorithm*.

Structure	Hemisphere	Measure	Resting State	Value
Frontal medial cortex	Left	Path length	RS3	96
Superior temporal gyrus	Right	Indegree	RS2	71
Frontal opercular cortex	Left	Clustering coefficient	RS2	68
Habenula nuclei	Right	Out strength	RS3	60
Cerebellum	Middle	Path length	RS2	53

*Boruta algorithm selected all the vital features from the entire feature space over 100 iterations based on random seeds. The number of times each feature is selected (value) represents the relative importance of the feature in the group classification.

Of note, for the young dataset, RS3 GI parameters (shown in blue) (largest component, lambda, and average shortest path) show up and support the separability. This indicates that the overall network topology changes stronger after food consumption than after visualization. In contrast, for the adult group, where no separation was found, nearly only node parameters are depicted, meaning changes are more specifically addressed to nodes, i.e., brain structures.

Table 3 shows the most relevant brain structures for the separation of AN vs. HCs in the young group, being the frontal medial cortex, most frequently selected in data separation, appearing in 96 out of 100 iterations.

## 4 Discussion

This study compared the impact of diverse sensory short-term modulations on RSNs through graph-theoretical and ML approaches in young and adult patients with AN vs. age-matched HCs. Overall, underlying RSNs of AN differed from HCs and additionally were differentially affected by visual stimulation (“anticipatory”) and food consumption (“consummatory”).

To summarize, the findings to the main questions showed baseline (no stimulation) RS connectivity alterations due to developmental differences in patients with AN vs. HCs. There were extensive developmental connectivity differences for the HCs when comparing young vs. adult, reflecting the natural RS development for HCs in contrast to very limited developmental maturation changes for AN. Short-term visual stimulation had an effect on AN and HC RSNs, particularly for the young cohort. Finally, the type of food consumption (low vs. high calorie) had a differential impact on RSNs, the cerebellum being particularly relevant during this process. The following discussion will deepen into each of these findings.

Patients with AN displayed significant differences in their RSNs for each age group. In both cases, the thalamus presented significantly different connectivity for AN vs. HCs. Previous studies have associated impaired thalamus connectivity with AN and its symptomatology regarding somatosensory/visual integration processes (Ehrlich et al., 2015; Geisler et al., 2016; Lord et al., 2016). Young participants showed more significant differences in the cerebellum and adults in the basal ganglia. Recently, neuroimaging attention has been redirected toward the cerebellum. Historically, most fMRI studies used to exclude this area, but it has been lately proven to play a much bigger role in higher-order functions than previously thought. The cerebellum seems to have a role in both feeding behavior and emotion regulation; studies showed the presence of different RS alterations in the cerebellar network in AN (Amianto et al., 2013; Sudo et al., 2024). In a previous study, we detected multiple seemingly non-specific activation differences in both directions with differential patterns in adolescents (mostly hypoactivation) and adults with AN (Horndasch et al., 2018). In young participants more acute starvation effects are likely; however, more research on differences between age groups is necessary to further elucidate developmental mechanisms. The finding of differences in basal ganglia connectivity is also consistent with other neuroimaging findings and have been linked to reward evaluation, reward-motivated decision-making, and controlling behavior (Via et al., 2021; Leppanen et al., 2020), which are likely to play a role in habit forming and maintenance of dysfunctional behavior and therefore could be present to a greater extent in more chronic, adult patients.

These disturbances in both AN age groups seemed to have been the result of impaired developmental differences caused by AN. Our NBS results showed widespread developmental connectivity differences for the HCs when comparing young vs. adults, corresponding to the natural RS development for HCs (Stevens et al., 2009). By contrast, only a few (most likely random) connectivity differences for AN were observed in networks involving the thalamus, basal ganglia, and frontal structures, all involved in mediating aspects of cognitive dysfunction in patients with AN (Gaudio et al., 2016). The AN cohort showed a whole brain generalized disturbed RS connectivity maturation. This is interesting in the context of a recent study showing in adolescent patients with AN—unlike our study—decreased functional connectivity in the DMN and two subcortical networks and linking the functional alterations to structural deficits in the sense of reduced cortical thickness. The authors conclude that restricted food intake in patients with AN can disrupt normal age-related brain maturation and the development of functional networks (Myrvang et al., 2021). However, to the best of our knowledge, until today no literature described the maturation disturbances in RS connectivity from adolescence to adulthood in AN. Furthermore, previously observed deep and enduring functional differences in brain organization could be an additional explanation for reduced RS connectivity in recovered restricted AN (Scaife et al., 2017; Gaudio et al., 2016).

In addition, our study revealed that the type of stimulation affected the RSNs, supporting the evidence of differential affective and motivational eating processes. This theory was particularly supported through our ML analysis for the young group where, in addition to global network topology, the frontal medial cortex, superior temporal gyrus, and frontal operculum were found vital for separation between stimulation modality in AN vs. HCs. These regions had already been associated with symptom-provoking paradigms (Uher et al., 2004), body image processing (Vidal et al., 2021), or taste processing (Frank et al., 2016). Our ML analysis was found unable to separate between conditions for the adult groups, which parallels our finding that only minor differences were found by all other statistical assessments (see above and below).

Visual stimulation by food items is associated with “anticipatory” neuronal mechanisms. Our cohort of young HCs revealed the strongest impact by this stimulation. Healthy young adolescents often prefer the flavors of high-calorie foods (Cooke and Wardle, 2005) without the concerns of the potential weight gain. Furthermore, our results showed how young patients with AN presented a diminished response to visual stimuli in comparison with their corresponding HCs, which is also manifested in the food desire ratings, where young patients with AN scored significantly lower than HCs (*p* < 0.001). Previous BOLD stimulation fMRI studies have associated stimulation with food images with increased cortical neural responses in adolescents and adults with AN in some brain regions, but diminished activation in others (Horndasch et al., 2018; Brooks et al., 2012). However, our results do not match the aforementioned findings given that RSNs, especially in adolescence, may be differentially affected by stimulus-driven fMRI responses, and still be showing and reflecting different underlying cognitive processing mechanisms toward food stimuli. As mentioned earlier, the fact that AN causes altered and few RS developmental changes could explain why these differences remain the same from young to adult age. Following this, adult and young individuals with AN may become desensitized to affective visual stimulation throughout the course of the disease [in the context of alexithymia and impaired emotional awareness in AN, see, e.g., (Horndasch et al., 2018)] and therefore present fewer RS changes by short-term stimulation. Studies have suggested sensitization-like changes in brain networks shaped by exposure to certain diets and restrictions that model oscillations between dieting and binging on palatable foods (Berridge, 2009). Regarding alcohol use disorder, previous research also demonstrated that the sensitization to affective visual stimuli as indicated by an attentional bias is moderated by the duration of the illness, cognitive function (Loeber et al., 2009), and the duration of abstinence (Vollstädt-Klein et al., 2009).

Altered RS consummatory mechanisms in AN were investigated here through food consumption stimulation. Our results evidence that the HC group presents similar RS outcomes to high-and low-calorie foods although they had more explicit anticipatory reactions during the rating for high-calorie foods. The small but significant differences between young and adult HCs could be explained by to the natural maturation of the brain previously discussed for the baseline differences. Furthermore, young and adult individuals with AN showed dramatically opposite connectivity responses to food consumption, particularly to high-calorie food consumption. Young individuals with AN presented significant network changes, differently affected by high-and low-calorie food consumption. In contrast, adults with AN showed no significant differences in network changes for the two conditions. These findings can support the theory of RS desensitization toward food stimuli with aging, being present during the “anticipatory” RS and the “consummatory” mechanisms. This desensitization can at least partly be explained as a consequence of the structural atrophy caused by the disease (Scharner and Stengel, 2019; Fonville et al., 2014) and therefore poor maturation of the RSNs, which, in turn, could contribute to the aberrant eating behaviors and the high chronicity of the disease. In addition, the results showed once again that the cerebellum plays an important role in feeding behaviour in young patients with AN, and to a considerable extent after food intake (consummatory mechanisms). These results contrast previous literature where adult patients with AN show for high-calorie stimuli stronger but for low-calorie stimuli weaker cerebellum activity than young patients (Horndasch et al., 2018). Again, this could be due to the fact that previous studies did not focus on the impact on the RSN connectivity changes and did not distinguish between the two different reward mechanisms involved in food consumption.

Several limitations of the current study should be acknowledged. The study was carried out by administering two specific types of food, chips and zucchini. Potato chips were chosen as they are considered one of the most “addictive” foods (Schulte et al., 2015) and—as opposed to, e.g., pizza or ice cream—can be easily administered in the experimental setting. Regarding desire ratings, the variation between participants was relatively low, but, of course, food preferences in general vary and individual differences could not be considered. The length of the disease or first onset was not collected. This measure could be interesting to see whether the RS maturation changes are more accentuated in patients with a longer disease history. Moreover, the time since hospitalization was also not registered. As all measurements took place at the beginning of treatment and no longitudinal data for the same person were acquired, no conclusions can be made about whether the RS effects could be reversed after weight restoration or intensive therapy treatment. Finally, note that the average BMI for adults with AN was 17.1, not extremely underweight. Due to the estradiol standard clinical analysis routine, we were only able to see quantitative values above 5.0 (pg/mL). Given that this procedure is not sensitive enough, results could only show a trend toward significant differences (adult *p* = 0.11; young *p* = 0.06)—this could be improved with further technical advanced analysis. This more in-depth analysis has the potential to reveal significant differences between groups and might serve as a disease biomarker. Furthermore, recent studies have been trying to use estrogen stimulation to potentially improve bone mineral density in AN (Resulaj et al., 2020).

The results from this study contribute to elucidating how AN plays a role in the maturation of the RSNs of the brain and how this could be a maintaining factor for the longevity and chronicity of the disease. Furthermore, it is of great importance to understand and study the nature of the food-related brain reward system while considering its different mechanisms, which are distinctively affected by AN. Further research involving longitudinal approaches and the inclusion of recovered AN participants could help to understand the predictive value of these brain connectivity changes for long-term recovery.

### 4.1 Conclusion

This study supports the hypothesis of differential reward mechanisms for food “anticipatory” and “consummatory” responses in AN. Additionally, baseline RS connectivity showed minimal maturation changes from young to adult patients with AN in comparison with HCs. The RS manifestations for the different reward mechanisms seem to be determined by the developmental stage of the individual. The ‘consumptive’ mechanism shows a high RSN reactivity in younger patients with AN, especially with high-calorie foods. This RSN reactivity decreases with age and is no longer present in adult patients with AN. This is probably due to desensitization that takes place after years of stimulus inhibition together with structural atrophy (Boto et al., 2017) and to the poor maturation of the RSNs, which could serve as a maintaining factor for the aberrant eating behaviors in individuals with AN, and therefore contribute to the high chronicity of the disease.

## Data Availability

The raw data supporting the conclusions of this article will be made available by the authors, without undue reservation.
