# Supplementary material for: Impaired maturation of resting-state connectivity in anorexia nervosa from adolescence to adulthood: differential mechanisms of consummatory vs. anticipatory responses through a symptom provocation paradigm
Source: Front Behav Neurosci. 2024 Oct 24;18:1451691. doi: 10.3389/fnbeh.2024.1451691 (PMC11541234; doi:10.3389/fnbeh.2024.1451691)
Supplement: Supplementary file 1 [file Table_1.DOCX]

Supplementary Material

Impaired Resting State Connectivity maturation in anorexia nervosa from adolescence to adulthood and differential consummatory vs anticipatory mechanisms through symptom provocation paradigm.

Andrea Mendez-Torrijos ^1^†, Mageshwar Selvakumar ^1^†, Silke Kreitz ^1,2^, Julie Roesch ^2^ , Arnd Dörfler^2^, Georgios Paslakis ^3^, Johannes Krehbiel ^4^, Sabine Steins-Löber ^5^, Oliver Kratz ^6^, Stefanie Horndasch ^6^†, Andreas Hess ^1,2,7^†

*** Correspondence:**Prof. Dr. Andreas Hess / Phone: +49 9131-85-22003 / Email: andreas.hess@fau.de

# Supplementary Figures and Tables

#
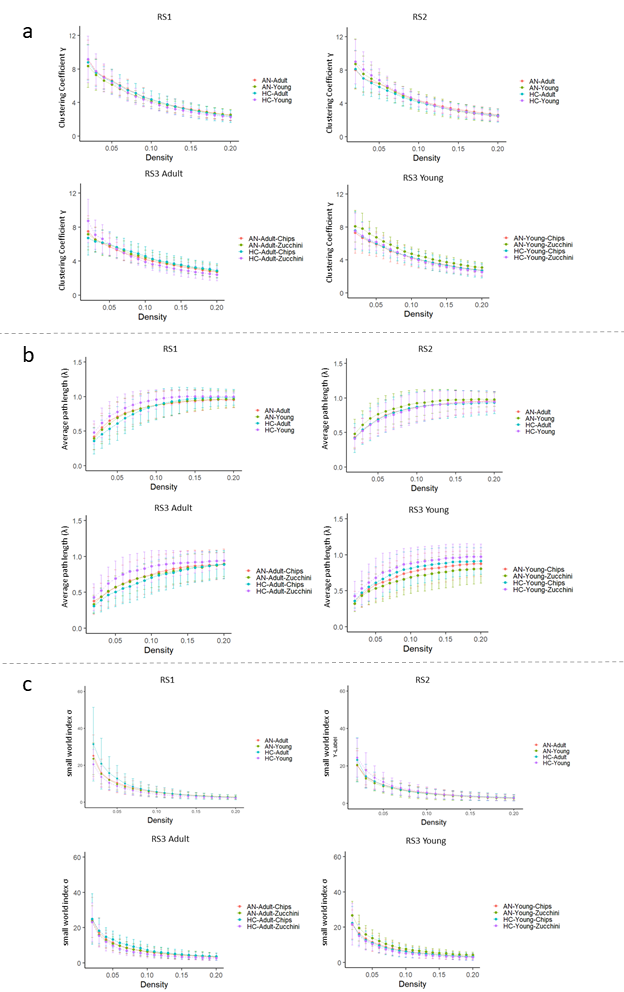


**Supplementary Figure 1.** General topology parameters for the different RS networks. Clustering coefficient (ɣ)(a) and average path length (λ)(b) were normalized to the mean of 10000 random networks with the same number of edges and nodes. The small world index (c) is the quotient of γ/λ. No significance was found between groups.

**Supplementary Table 1.** Hypothesis and results from NBS

| **Hypothesis** | **NBS Testing** | **Type of NBS** | **pFWE/ Alpha (α)** | **Nodes** | **Connections** |
| --- | --- | --- | --- | --- | --- |
| Base a): Are there RS connectivity alterations in AN brains? | $\Delta_{HC-AN}A_{M}{01}_{RS}1$ | Homo | 0.03/0.05 | 177 | 381 |
|  | $\Delta_{HC-AN}Y_{M}{01}_{RS}1$ |  | 0.04/0.05 | 126 | 349 |
| Base b): Are there RS connectivity developmental differences? | $\Delta_{A-Y}{HC}_{M}{01}_{RS}1$ | Homo | 0.01/0.05 | 150 | 378 |
|  | $\Delta_{A-Y}{AN}_{M}{01}_{RS}1$ |  | 0.42/0.05 | 72 | 91 |
| Visual a): Is there an impact of visual stimulation on AN RSNs? | $\Delta_{RS2-RS1}A$ | - | - | - | - |
|  | $\Delta_{RS2-RS1}Y$ |  | - | - | - |
| Food a) & b): Is there an effect after eating chips/zucchini within AN age groups? | $\Delta_{RS3-RS2}{A_{Ch}}_{AN-HC}$ | -Paired  -Control: RS3-RS2 HC | 1/0.01 | 1 | 0 |
|  | $\Delta_{RS3-RS2}{Y_{Ch}}_{AN-HC}$ |  | 0.00/0.00 | 103 | 703 |
|  | $\Delta_{RS3-RS2}{A_{Zuc}}_{AN-HC}$ |  | 0.03/0.02 | 99 | 500 |
|  | $\Delta_{RS3-RS2}{Y_{Zuc}}_{AN-HC}$ |  | 0.00/0.01 | 111 | 468 |
| Food c): Which brain structures selectively respond to high vs low-calorie food consumption? | $\Delta_{RS3-RS2}{A_{HC}}_{Ch-Zuc}$ | -Paired  -Control: RS3- RS2 Zucchini | 0.9/0.01 | 38 | 42 |
|  | $\Delta_{RS3-RS2}{Y_{HC}}_{Ch-Zuc}$ |  | 0.1/0.01 | 41 | 51 |
|  | $\Delta_{RS3-RS2}{A_{AN}}_{Ch-Zuc}$ |  | 1/0.00 | 1 | 0 |
|  | $\Delta_{RS3-RS2}{Y_{AN}}_{Ch-Zuc}$ |  | 0.09/0.00 | 83 | 245 |

Single connections are significance is represented by alpha (uncorrected for multiple comparisons). pFWE is the significance (as a whole component) after testing for multiple comparison. pFWE above 0.1 means that there is a chance higher than 10% that a component of the observed size occurs randomly. There is no explicit convention for the threshold of the pFWE, for this we accept a chance of 10 % of random occurrence. (Homo= Homoscedastic, Ch=chips, Zuc= Zucchini).
